# Complete genome sequence of *Erwinia amylovora* PBI209 isolated from a necrotic flower of *Pyrus sinkiangensis* in China

**DOI:** 10.1128/mra.00291-24

**Published:** 2024-07-05

**Authors:** Ya Gao, Bo Song, Meihong Wang, Fenghuan Yang, Chao Yu, Huamin Chen

**Affiliations:** 1 State Key Laboratory for Biology of Plant Diseases and Insect Pests, Institute of Plant Protection, Chinese Academy of Agricultural Sciences, Beijing, China; 2 Institute of Plant Protection, Xinjiang Academy of Agricultural Sciences, Urumqi, China; 3 Key Laboratory of Integrated Pest Management on Crops in Northwestern Oasis, Ministry of Agriculture and Rural Affairs, Urumqi, China; 4 Xinjiang Key Laboratory of Agricultural Biosafety Urumqi, Urumqi, China; The University of Arizona, Tucson, Arizona, USA

**Keywords:** *Erwinia amylovora*, fire blight

## Abstract

Here, we report the complete genome sequence of *Erwinia amylovora* PBI209 that causes fire blight isolated from a necrotic flower of *Pyrus sinkiangensis* in Xinjiang, China. The genome consists of 3,800,955 bp, with 3,403 protein-coding genes and a guanine-cytosine content of 53.61%.

## ANNOUNCEMENT

*Erwinia amylovora* is an economically devastating plant pathogen that causes fire blight disease in pear, apple, and other plants in the *Rosaceae* family (1, 2). It was the first bacterial plant pathogen described and originated in North America, from where it has spread to nearly 60 countries and regions (3, 4). In China, fire blight was first discovered in Xinjiang Uygur Autonomous Region in 2016 (5). In this study, we isolated *E. amylovora* PBI209 from a necrotic flower of *Pyrus sinkiangensis* in Korla (41°48′53″N, 85°49′53″E), Xinjiang Uygur Autonomous Region, China. The infected flower was sterilized and ground in ddH_2_O with a mortar and grinding rod, and the mixture was diluted to optimal concentration and spread onto nutrient agar (NA) plates. The strain PBI209 colonies were selected after incubation at 28°C for 36 h and identified as *E. amylovora* by sequences of 16S-rDNA fragments (6) and Koch’s rule (7).

For genomic sequencing, a single colony of the PBI209 strain was picked and cultured in 50-mL NA medium at 28°C until the culture reached an optical density of 1.0 at 600 nm (optical density at 600 nm), then the cells were harvested by centrifugation for 10 min at 6,000 × *g*. Genomic DNA was extracted using magnetic bead-based extraction method (8) and quantified with Qubit (v.3.0) fluorimeter (Life Technologies, Carlsbad, CA, USA). The obtained genomic DNA was sequenced using a Pacbio Sequel II platform at the Beijing Genomics Institute (Shenzhen, China). Four single molecule real-time cell Zero-Mode Waveguide arrays of sequencing were used to generate the subreads set, and subreads below 1,000 bp were removed. Then subread self-correction and genome assembly were performed using Canu (v.1.5) (9). To improve the accuracy of the genome sequences, GATK (v.1.6–13) was used to make single-base corrections. A total of 157,332 subreads with an *N*_50_ length of 27,495 bp and an average read length of 17,297 bp were obtained through PacBio sequencing, which provided approximately 330-fold genome coverage. Gene prediction was performed on the *E. amylovora* genome assembly by glimmer (v.3.02) with hidden Markov models (10), and gene general function was annotated by blasting genes with different databases using Diamond software (11 – 16). Default parameters were used for all software unless otherwise specified.

The complete genome of PBI209 contains a circular chromosome of 3,800,955 bp with a GC content of 53.61% and a plasmid of 28,257 bp with a GC content of 50.24%, and the prediction of genome resulted in 3,403 genes, 22 rRNAs, 77 tRNAs, and 32 sRNA (Table 1). In addition, the genome of PBI209 has high sequence identity with the genome of CFBP1430 (17) and 99east-3–1 (18), with an average nucleotide identity (ANI) of 99.98% and 99.94%, respectively. The ANI genome comparisons were performed using EzBioCloud (https://www.ezbiocloud.net/tools/ani) (6).

**TABLE 1:** *E. amylovora* PBI209 genomic features^*a*^

Genomic features		Number
Genome project information	GenBank accession number	CP157669-CP157670
	BioProject accession number	PRJNA1089544
	BioSample accession number	SAMN40544208
	SRA accession number	SRR28393279
PacBio data	Total read length (bp)	2,721,481,689
	Subreads	157,332
	Maximum subread length (bp)	158,832
	Average subread length (bp)	17,297
	Minimum subread length (bp)	2,000
	*N*_50_ read length (bp)	27,495
	*N*_90_ read length (bp)	8,315
Genome assembly statistics	Size (bp)	3,800,955
	GC%	53.61
	Genes	3,403
	rRNAs (5S, 16S, and 23S)	8, 7, and 7
	sRNA	32
	tRNA	77
	Pseudogenes	82
	CRISPR arrays	4

^*a*^ CRISPR, clustered regularly interspaced short palindromic repeats; SRA, sequence read archive.

## Data Availability

The whole-genome sequencing project of PBI209 has been deposited at NCBI GenBank under the accession number CP157669 and CP157670 BioProject accession number PRJNA1089544 and BioSample accession number SAMN40544208. The raw sequences have been deposited in the SRA under the accession number SRR28393279.
